# miR-29a Modulates Neuronal Differentiation through Targeting REST in Mesenchymal Stem Cells

**DOI:** 10.1371/journal.pone.0097684

**Published:** 2014-05-19

**Authors:** Ping Duan, Shiling Sun, Bo Li, Chuntian Huang, Yan Xu, Xuefei Han, Ying Xing, Wenhai Yan

**Affiliations:** 1 Institute of Basic Medicine, Zhengzhou University, Zhengzhou, Henan, China; 2 Hematology Department in the First Affiliated Hospital of Henan University of Traditional Chinese Medicine, Zhengzhou, Henan, China; 3 Department of Physiology, Xinxiang Medical University, Xinxiang, Henan, China; University of Torino, Italy

## Abstract

**Objective:**

To investigate the modulation of microRNAs (miRNAs) upon the neuronal differentiation of mesenchymal stem cells (MSCs) through targeting RE-1 Silencing Factor (REST), a mature neuronal gene suppressor in neuronal and un-neuronal cells.

**Methods:**

Rat bone marrow derived–MSCs were induced into neuron-like cells (MSC-NCs) by DMSO and BHA *in vitro*. The expression of neuron specific enolase (NSE), microtubule-associated protein tau (Tau), REST and its target genes, including synaptosomal-associated protein 25 (SNAP25) and L1 cell adhesion molecular (L1CAM), were detected in MSCs and MSC-NCs. miRNA array analysis was conducted to screen for the upregulated miRNAs after neuronal differentiation. TargetScan was used to predict the relationship between these miRNAs and REST gene, and dual luciferase reporter assay was applied to validate it. Gain and loss of function experiments were used to study the role of miR-29a upon neuronal differentiation of MSCs. The knockdown of REST was conducted to show that miR-29a affected this process through targeting REST.

**Results:**

MSCs were induced into neuron-like cells which presented neuronal cell shape and expressed NSE and Tau. The expression of REST declined and the expression of SNAP25 and L1CAM increased upon the neuronal differentiation of MSCs. Among 14 upregulated miRNAs, miR-29a was validated to target REST gene. During the neuronal differentiation of MSCs, miR-29a inhibition blocked the downregulation of REST, as well as the upregulation of SNAP25, L1CAM, NSE and Tau. REST knockdown rescued the effect of miR-29a inhibition on the expression of NSE and Tau. Meanwhile, miR-29a knockin significantly decreased the expression of REST and increased the expression of SNAP25 and L1CMA in MSCs, but did not significantly affect the expression of NSE and Tau.

**Conclusion:**

miR-29a regulates neurogenic markers through targeting REST in mesenchymal stem cells, which provides advances in neuronal differentiation research and stem cell therapy for neurodegenerative diseases.

## Introduction

Mesenchymal stem cells (MSCs) are multipotent mesoderm-derived somatic stem cells that are present in the stroma of virtually all mammalian organs [1]. Previous studies indicate that MSCs can differentiate into osteogenic, chondrogenic, adipogenic, myogenic, fibroblastic, neuronal lineages [2]–[4]. The ability to differentiate MSCs in vitro toward a neural lineage could have potential therapeutic applications in the treatment of neurological diseases and CNS trauma. Numerous studies suggest that MSCs could produce mature neuron-like cells that exhibit multiple neuronal properties and traits, such as action potential, synaptic transmission, secretion of neurotrophic factors and dopamine, and spontaneous postsynaptic current [5]–[12]. However, the mechanisms of neuronal differentiation from MSCs remain elusive.

Repressor element-1 silencing transcription factor (REST), also known as neuronal restrictive silencing factor (NRSF), is a transcriptional regulator that can repress a battery of neuronal differentiation genes in non-neuronal cells or in neural cells [13]–[15]. REST activity is reduced as progenitors differentiate into neurons, allowing the expression of neuronal genes [16]–[18]. Downregulation of REST during neurogenesis is necessary for proper neuronal differentiation, and overexpression of REST in differentiating neurons disrupts neuronal gene expression and causes axon guidance errors [19]. Previous reports have shown that REST can modulate the neuronal differentiation of MSCs. Yang et al [20] demonstrated that REST silencing activates a multiple neuron-specific genes including brain-derived neurotrophic factor (BDNF), neurogenin 1 (NGN1), neuron-specific enolase (NSE), synaptophysin (SYP), and neuron-specific growth-associated protein (SCG10), and induces neuronal differentiation of MSCs. Trzaska et al [21] showed that the loss of REST in MSC-derived dopamine progenitors induces functional maturity, suggesting that REST is a limiting gene in the generation of functional mature neurons from MSCs. Although Trzaska et al [21] did not detect significantly change of REST expression upon the neuronal differentiation of MSCs, Liu et al [22] found that REST mRNA expression declines, and accompanied with that, REST-related genes are upregulated. The inconsistent results of REST expression may come from different induction means.

miRNAs comprise a group of small (19–25 nt) non-coding RNAs that regulate gene expression by binding to their target messenger RNAs (mRNAs), thus resulting in translational repression or mRNA degradation [23]–[25]. Specific miRNAs have been shown to be involved in the neurogenesis of MSCs. MiR-9 promotes the neural differentiation of mouse bone marrow MSCs via targeting zinc finger protein 521 [26]. miR-130a and miR-206 inhibit neurotransmitter substance P (SP) releasing through targeting TAC1 in MSC-derived neuronal cells [27]. miR-34a contributes in the motility of MSCs-derived neural precursors [28].

In the present study, we hypothesized that the expression of REST was regulated by miRNAs upon the neurogenesis of MSCs. We conducted miRNA array to analyze the differential expression of miRNAs between MSCs and MSC-derived neuron-like cells (MSC-NCs). Then we demonstrated that miR-29a modulates neuronal differentiation through targeting REST in MSCs.

## Materials and Methods

### Ethics statement

The animal studies were approved by the Animal Ethics Committees of Zhengzhou University. Animals were housed and treated under the approved protocols. All rat work was consistent with the requirement of the Animal Ethics Committees of Zhengzhou University. All efforts were made to minimize animal suffering.

### Cell isolation and cultivation

Cells harvested from the femurs and tibias of Sprague-Dawley rats (Laboratory Animal Center, Zhengzhou University, China) were originally cultured in Dulbecco's modified Eagle's medium (DMEM; Gibco, Grand Island, NY, cat. no. 12320–032) supplemented with 10% fetal bovine serum (FBS; Gibco, cat. no. 16000–036), 100 U/ml penicillin, and 100 mg/ml streptomycin. For each passage, the cells were plated at about 8,000 cells/cm^2^ and grown to confluence.

### Neural induction of MSCs

Neural induction of MSCs was performed as described [4]. At passage 4, subconfluent cultures of rat MSC were maintained in DMEM/10% FBS. Twenty-four hours prior to neuronal induction, media were replaced with a preinduction media consisting of DMEM/20% FBS/1 mM β-mercaptoethanol (BME; Sigma, St. Louis, MO, cat. no. M3148). To initiate neuronal differentiation, the preinduction media were removed, and the cells were washed with PBS and transferred to neuronal induction media composed of DMEM/10 mM BME. In later experiments, DMEM/2% dimethylsulfoxide (DMSO; Sigma, cat. no. D8418)/200 mM butylated hydroxyanisole (BHA; Sigma, cat. no. B1253) was utilized as the neuronal induction media. The percentage of cells with neuron-like morphology was calculated in 10 randomly chosen fields under inverted microscope. Cells were fixed for immunocytochemistry at 5 h postinduction. The data analyzed were from 3 independent experiments.

### Immunocytochemistry

Cultured MSCs and MSC-NCs were fixed with 4% paraformaldehyde, incubated with primary antibody overnight at 4°C, and incubated with secondary antibody for 1 h, followed by exposure to avidin–biotin complex for 1 h (25°C). 3, 3'-Diaminobenzidine (DAB, Sigma, cat. no. D8001) served as the chromogen. The antibodies against neuron specific enolase (NSE, Santa Cruz Biotechnology, Santa Cruz, CA, cat. no. sc-7455) and microtubule associated protein tau (Santa Cruz, cat. no. sc-5587) were used at a 1∶100 dilution.

### Western blot

Western blot analyses were performed as described [29]. Antibodies against NSE (Santa Cruz, cat. no. sc-7455), Tau (Santa Cruz, cat. no. sc-5587), REST (Santa Cruz, cat. sc-25398), and β-actin (Santa Cruz, cat. no. sc-47778) were diluted 1∶1,000. Secondary antibodies were goat anti-mouse IgG-HRP (Santa Cruz, cat. no. sc-2005) or goat anti-rabbit IgG-HRP (Santa Cruz, cat. no. sc-2004). Enhanced chemiluminescence was performed according to the manufacturer's instructions (Amersham Life Sciences Inc., Arlington Heights, IL).

### Microarray

Cells acquired from three independent experiments were mixed for microarray assay. Total RNA was isolated using TRIzol (Invitrogen, Carlsbad, CA, USA) and miRNeasy Mini Kit (Qiagen, Hilden, Germany) according to the manufacturer's instructions. After RNA isolation from the samples, the miRCURY Hy3/Hy5 Power labeling kit (Exiqon, Vedbaek, Denmark) was used according to the manufacturer's guidelines for miRNA labeling. The Hy3™-labeled samples were hybridized on the miRCURYTM LNA Array (v.14.0) (Exiqon) according to the array manual. Hybridized DNA microarrays were scanned with the Axon GenePix 4000B microarray scanner (Axon Instruments, Foster City, CA). Scanned images were then imported into GenePix Pro 6.0 software (Axon) for grid alignment and data extraction. Replicated miRNAs were averaged, and miRNAs with intensities greater than 50 in all samples were used in calculation of the normalization factor. Expressed data were normalized using median normalization. After normalization, differentially expressed miRNAs were identified through fold change filtering.

### Quantitative RT-PCR analysis (qRT-PCR)

Total RNA, including miRNAs, was extracted using mirVana miR isolation kit (Ambion) in accordance with the manufacturer's instructions. miR-29a/b, miR-294, and miR-291-5p were detected by using RT^2^ miRNA First Strand Kit (SA biosciences), and specific miRNA and U6 primers from QIAGEN were used for real-time PCR. Relative expression was calculated using the comparative Ct method (2^−[Δ] [Δ] Ct^).

Expression of mRNAs was determined using SYBR green real-time PCR assay. The PCR primers used were as follows: 5′-ACTCGACACATGCGTACTCACTCA-3′ and 5′-CTTGCGTGTCGGGTCACTTC-3′ (REST, NM_031788.1); 5′-AGCCCAAAGACTCCTCCA-3′ and 5′-TGCTGTAGCCGCTTCGTTCT-3′ (Tau, NM_017212.2); 5′-TCGCCACATTGCTCAACT-3′ and 5′-AACTCAGAGGCAGCCACATC-3′ (NSE, NM_139325.3); 5′-TGTCTTGGAGCCCTGCTGAA-3′ and 5′-CTGGCACCTTGCCTAGACTGAAC-3′ (L1CAM, NM_017345.1); 5′-ACACCCAGAATCGCCAGATTG-3′ and 5′-TGCACGTTGGTTGGCTTCA-3′ (SNAP25, NM_001270575.1); 5′- AGATGGACAAGTTCCCCTTTG -3′ and 5′-ACACAAGTAGGCAGTGGCAGT-3′ (ALP, NM_013059.1); 5′-TTCAGCTCTGGGATGACCTT-3′ and 5′-TGCCACTCAGAAGACTGTGG-3′ (GAPDH). The levels of mRNA expression were normalized to that of GAPDH. Relative expression was calculated using the comparative Ct method (2^−[Δ] [Δ] Ct^).

### Constructs

luc-UTR vectors was constructed by cloning predicted miR-29a target region or its mutant control into the *Nhe*I and *Sal*I sites of the pmirGLO luciferase vector (Promega, Madison, WI) using the PCR generated fragments. The oligonucleotide pairs contain *Kpn*1 internal site for clone confirmation: sense-wt: 5′-**CTAGC**TAGGTACCTGGTATTTTTTACTTTGGTGCTT**G**-3′ and anti-sense-wt: 5′-**TCGAC**AAGCACCAAAGTAAAAAATACCAGGTACCTA**G-**3′; sense-mut: 5′-**CTAGC**TAGGTACCTGGT*GAAG*TTTACTT*GACGCGAT* **G**-3′ and anti-sense-mut: 5′-**TCGAC** *ATCGCGTC*AAGTAAA*CTTC*ACCAGGTACCTA**G**-3′. Bold indicates *Nhe*I and *Sal*I sites; underline indicates the *Kpn*1 site; italics indicates the mutated sites.

### Cell infection in MSCs

The lentiviral vectors containing miR-29a precursor (RmiR6139-MR03), scramble control (CmiR0001-MR03) were obtained from GeneCopoeia Inc. The lentivirus containing miR-29a inhibitor and its control was obtained from Baoke Bio-Technology Co., Ltd. (Zhengzhou, China).

Lentivirus was generated by using Lenti-Pac HIV Expression Packaging Kit (GeneCopoeia). For the transduction of MSCs with lentivirus, 1×10^6^ MSCs were plated, and 20 µl of virus suspension (at a MOI of 50) was added. The cells were placed for 2 h at 4°C, transferred to a plate, and cultured in 5% CO_2_ at 37°C for 4 d.

The siRNA lentivirus against REST and its control were obtained from Baoke Bio-Technology Co., Ltd. MSCs were transfected with miR-29a inhibitor for 4 d, then 1×10^6^ MSCs were plated, and 20 µl of virus suspension (at an MOI of 50) was added. The cells were cultured in 5% CO_2_ at 37°C for 2 d.

### Luciferase assay

293 cells were infected with lentivirus carrying miR-29a precursor for 2 d. Then the cells were transfected with pmirGLO-REST-wt, pmirGLO-REST-mut or pmirGLO-ctrl using Lipofectamine 2000 (Invitrogen). Luciferase activity was measured 24 h after transfection using the Dual-Glo luciferase assay system (Promega). The *Renilla* luciferase activity served as internal control.

### miRNAs target gene prediction

Computational analyses to predict potential binding between the 3′ UTR of target genes and miRNA were carried out with TargetScan (version 6.0).

### Statistical analysis

Statistical evaluation of data was performed using SPSS 12 analysis software (SPSS, Chicago, IL). Comparisons were made using the independent-samples t-test. The level of significance was set at *P*<0.05.

## Results

### The expression of REST declines with neuronal differentiation of MSCs

**Figure 1:**
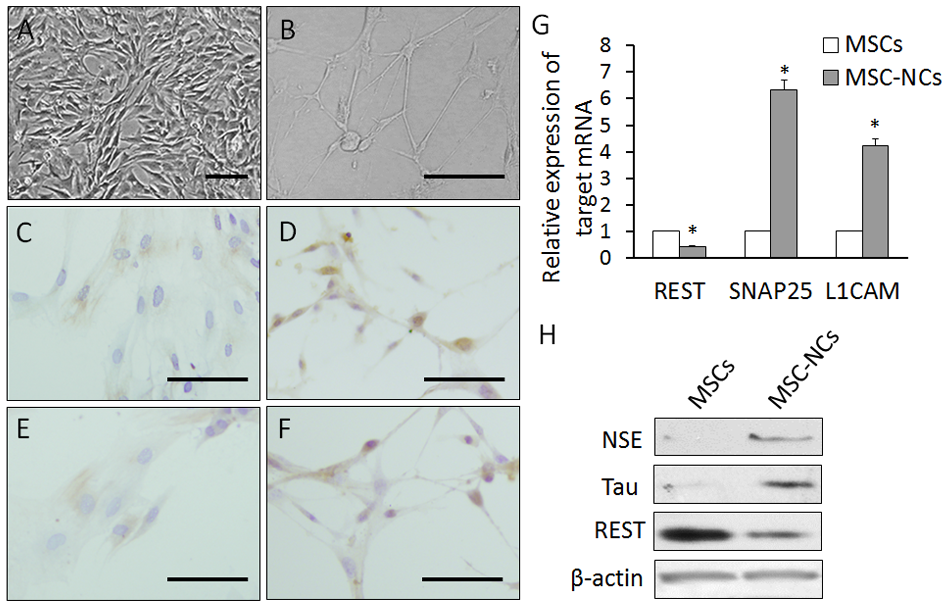
The expression of REST declines upon neuronal differentiation of MSCs. (A) MSCs before neuronal induction. (B) MSCs induced by DMSO/BHA for 5 h. About 80% of the cells adopted a neuron-like morphology, outgrowing long dendrites. (C) Immunostaining of NSE in MSCs. (D) Immunostaining of NSE in MSC-derived neuron-like cells (MSC-NCs). (E) Immunostaining of Tau in MSCs. (F) Immunostaining of Tau in MSC-NCs. Scale: 400 µm. (G) The mRNA expression of REST and its targets SNAP25 and L1CAM in MSCs and MSC-NCs detected by qRT-PCR. (H) Western blot results in MSCs and MSC-NCs. Mean ± SEM of 5 independent MSC cultures are shown. *: *P*<0.01, all compared with MSCs.

Previous experiments determined that DMSO and BHA could rapidly induce MSCs to differentiate into neuron-like cells [30], [31]. Using the same protocol, we induced rat bone marrow-derived MSCs. Subconfluent MSC cultures were changed to serum-free medium containing DMSO/BHA (induction medium). After 5 h, about 80% of the cells had adopted a neuron-like morphology, and expressed the neuronal markers NSE and Tau detected by immunostaining and western blot (Fig. 1A-F, H). Meanwhile, the mRNA and protein expression of REST was decreased in MSC-NCs when compared with that in MSCs (Fig. 1G, H). We also measured the expression of synaptosomal-associated protein 25 (SNAP25) and L1 cell adhesion molecular (L1CAM), which are direct target genes of REST and negatively regulated by REST [32]. The results showed that the expression of SNAP25 and L1CAM was increased with the downregulation of REST upon the neuronal differentiation of MSCs (Fig. 1G).

### miRNA expression change with neuronal differentiation of MSCs

**Figure 2:**
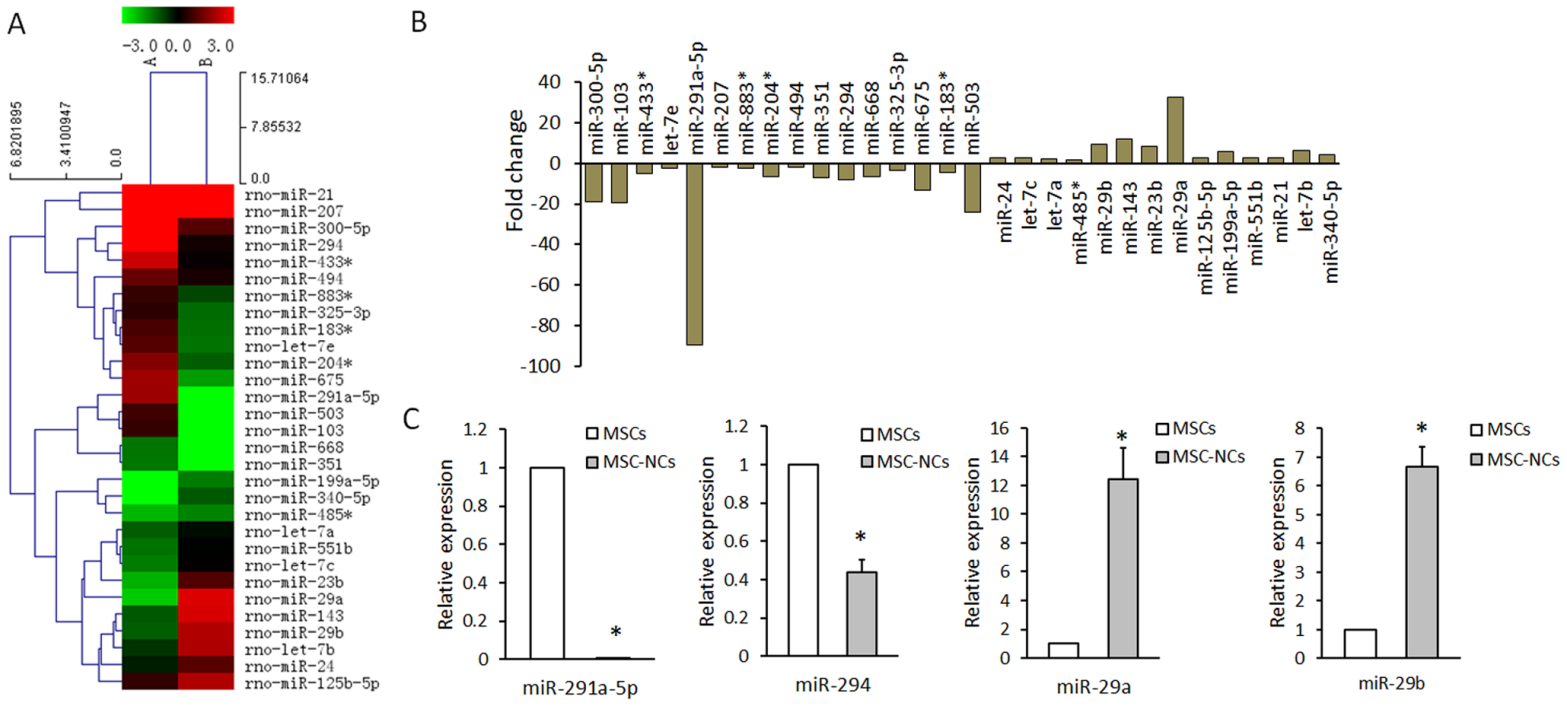
The differential expression of miRNAs upon the neuronal differentiation of MSCs. (A) miRNA arrays of MSCs and MSC-NCs calculated using Cluster 3.0. The expression change indicated by the color bar was calculated by comparing the miRNA expression levels in MSC-NCs with that in MSCs. Only entire differentially expressed miRNAs pass fold change filtering (≥1.5-fold change). (B) Histogram of miRNA array results. Total RNA was extracted from MSC mixture or MSC-NC mixture from 3 independent experiments. Each group was detected once with miRNA array. (C) qRT-PCR results of miR-291a-5p, miR-294, miR-29a and miR-29b expression in MSCs and MSC-NCs. Mean ± SEM of 3 independent MSC cultures are shown. *: *P*<0.01, all compared with MSCs.

miRNA array was conducted to identify the differentially expressed miRNAs upon the neuronal differentiation of MSCs. Total RNA was extracted from MSC mixture or MSC-NC mixture from 3 independent experiments. Each group was detected once with miRNA array. We performed a fold change filtering between the MSCs and MSC-NCs (≥1.5-fold change). MicroRNA arrays showed that 16 miRNAs were downregulated and 14 miRNAs were upregulated in MSC-NCs when compared with those in MSCs (Fig. 2A, B). Among those differently expressed microRNAs, miR-291a-5p, mir-294, miR-29a, and miR-29b were further detected by qRT-PCR, and the results were consistent with the microRNA array analysis (Fig. 2C).

### REST is a direct target of miR-29a

**Figure 3:**
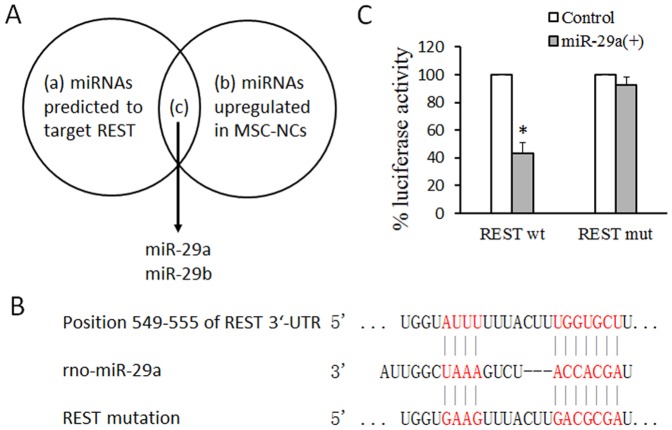
REST is a direct target of miR-29a. (A) miR-29a and miR-29b are predicted to target REST upon the neuronal differentiation of MSCs. (B) Predicted REST 3′-UTR binding sites for miR-29a. The alignment of the seed regions of miR-29a with REST 3′ UTR are shown. (C) REST is a target of miR-29a. pmirGLO luciferase construct containing a wild type (wt) or mutated (mut) REST 3′-UTR was co-transfected with miR-29a precursor or scramble control in 293 cells and the luciferase assay was performed. Mean and SEM of 3 independent MSC cultures are shown. *: *P*<0.01, compared with Control.

We supposed that miRNAs might regulate the expression of REST upon the neuronal differentiation of MSCs. Among the 14 miRNA upregulated in MSC-NCs, rno-miR-29a and rno-miR-29b were predicted to target REST analyzed by TargetScan (http://www.targetscan.org/) (Fig. 3A). A potential binding site was found in the 3′-UTR of REST at position 549-555 (Fig. 3B). Dual luciferase reporter assay was applied to validate the relationship between miR-29a and REST. The REST 3′-UTR containing the miR-29a binding site and its mutant were cloned into the pmirGLO vector downstream of the luciferase ORF, respectively. The vectors, including REST-wt, REST-mut and control, were transfected into miR-29a lentivirus-infected 293 cells in which miR-29a was overexpressed, respectively. Luciferase assay results showed that the luciferase activity was significantly inhibited in REST-wt-transfected cells compared with that in control (Fig. 3C). However, the luciferase activity did not change in REST-mut-transfected cells compared with that in control (Fig. 3C).

### miR-29a modulates neuronal differentiation of MSCs through targeting REST

**Figure 4:**
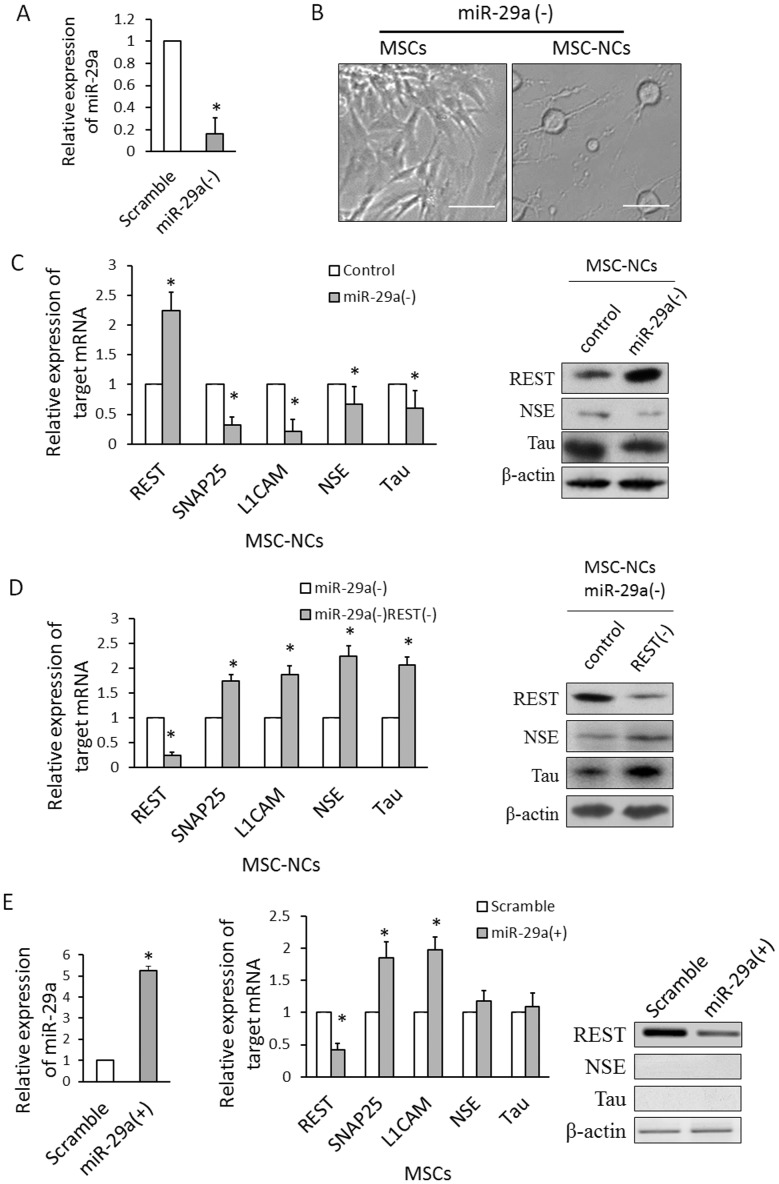
miR-29a modulates neuronal differentiation of MSCs by targeting REST. (A) Lentiviral infection of miR-29a inhibitor decreases miR-29a expression in MSCs. (B) Cell morphology of MSCs with miR-29a inhibition before and after neuronal induction. MSCs with miR-29a knockdown presents neuron shape after induction. (C) The expression of REST, SNAP25, L1CAM, NSE and Tau was detected by qRT-PCR and western blot in MSC-NCs with miR-29a inhibition. REST gene was upregulated by miR-29a inhibition. Meanwhile, SNAP25, L1CAM, NSE and Tau genes were downregulated by miR-29a inhibition. (D) The expression of REST, SNAP25, L1CAM, NSE and Tau was detected by qRT-PCR and western blot in MSC-NCs co-transfected with miR-29a inhibition and REST siRNA. NSE and Tau genes were upregulated by REST knockdown in MSC-NCs with miR-29a inhibition. (E) The mRNA and protein expression of REST, NSE, and Tau was detected in MSCs transfected with miR-29a precursor. REST was downregulated, and SNAP25 and L1CAMby were upregulated by miR-29a knockin. The expression of NSE and Tau was not significantly changed by miR-29a knockin. Scale: 400 µm. Mean ± SEM of 5 independent MSC cultures are shown. *: *P*<0.05, compared with control.

In order to study the role of miR-29a upon the neurogenesis of MSCs, we first conducted loss of function experiment. MSCs were infected with lentivirus containing miR-29a inhibitor or control. After 4 d, about 90% MSCs were successfully infected and expressed EGFP detected by Flow cytometer. miR-29a expression was significantly decreased after lentiviral infection of miR-29a inhibitor (Fig. 4A). Then these MSCs were induced into neuron-like cells (Fig. 4B). miR-29a knockdown increased the expression of REST and decreased the expression of SNAP25, L1CAM, NSE and Tau in MSC-NCs (Fig. 4C). We supposed that the miR-29a might regulate the expression of NSE and Tau through targeting REST gene during the neuronal differentiation of MSCs. Therefore, we used the lentivirus containing siRNA against REST to co-infect MSCs with miR-29a inhibitor. The expression of REST was significantly decreased after siRNA infection (Fig. 4D). In MSC-NCs with miR-29a inhibition, REST knockdown increased the expression of NSE and Tau (Fig. 4D). The arrest of NSE and Tau expression induced by miR-29a inhibition was partially blocked by REST knockdown, indicating that miR-29a regulated the expression of NSE and Tau through targeting REST gene.

We also conducted gain of function experiment by lentiviral transfection of miR-29a precursor in MSCs. The forced expression of miR-29a decreased the expression of REST and increased the expression of SNAP25 and L1CAM (Fig. 4E). However, single miR-29a knockin did not significantly upregulate NSE or Tau gene (Fig. 4E).

## Discussion

In the present study, we demonstrated that REST is downregulated upon the neuronal differentiation of MSCs. After analyzing miRNAs profiles in MSCs and MSC-NCs, we found that the expression of miR-29a increases during this process. We further showed that miR-29a modulates neuronal differentiation through targeting REST in MSCs.

We demonstrated that the mRNA and protein expression of REST is decreased when MSCs are induced into MSC-NCs by simple chemical means, a combination of DMSO and BHA. Liu et al [22] also found the decrease of REST mRNA in MSCs induced by another combination of beta-mercaptoethanol (β-ME) and DMSO. Although chemicals was extensively used to induce the neuronal differentiation of MSCs [31], [33], the underlying mechanism is still elusive. Our results have showed that the downregulation of REST might be one of the mechanisms to regulate the neuronal differentiation of MSCs upon this process.

We then showed that the downregulation of REST is modulated by miR-29a. The human miR-29 family of microRNAs has three mature members, miR-29a, miR-29b, and miR-29c. Mature miR-29s are highly conserved in human, mouse, and rat, and share identical sequences at nucleotide positions 2-7. miR-29s are involved in myogenic differentiation [34], skeletal myogenesis [35], osteoblast differentiation [36], [37], and neuronal differentiation [38], [39]. miR-29a/b-1 express in primary cultures of neuronal and glial cells [39]. We are the first one to report the upregulation of miR-29a/b in MSC-NCs. Considering that miR-29a and miR-29b have overlapped predicted target genes and the expression change of miR-29a is more outstanding, we chose miR-29a for further studies. We found that forced expression of miR-29a decreased REST expression in MSCs through directly targeting REST, indicating the potential role of miR-29a to modulate the neurogenesis of MSCs.

The present study demonstrated that miR-29a modulates the neuronal differentiation of MSCs. miR-29a affects the expression of NSE and Tau through directly targeting REST. It is remarkable that miR-29a mainly affects the expression of neuronal markers, but not the cell shape in MSCs after neuronal induction (Figure S1). We owe it to chemical induction method used in the present study, which may induce rapid morphological changes in various cell types, independent of neurogenesis [40]. However, it is still possible that miR-29a affects the cell morphology of MSCs upon neuronal differentiation using other means.

It is noteworthy that the exogenic expression of miR-29a did not effectively initiate the neuronal differentiation of MSCs, although it decreased the expression of REST gene. We detected the expression of NSE and Tau, both are neuronal specific markers, and found it was not significantly increased by miR-29a over-expression. Meanwhile, the cell morphology was not induced into neuron shape. In previous study, knockdown of REST by siRNA induces MSCs into neuronal cells, which exhibited neuron-like morphology and expressed multiple neuron-specific genes including brain-derived neurotrophic factor (BDNF), neurogenin 1 (NGN1), neuron-specific enolase (NSE), synaptophysin (SYP), and neuron-specific growth-associated protein (SCG10), as well as expressing mature neuronal marker proteins, such as β-tubulin III, NSE, microtubule-associated protein type 2 (MAP-2), and neurofilament-200 (NF-200) [20]. However, the present study showed that downregulation of REST by miR-29a overexpression did not increased the expression of NSE and Tau. Kapinas et al [41], [42] have shown that miR-29a promotes osteoblast differentiation. Therefore, we detected the expression of alkaline phosphatase (ALP), a marker of osteogenesis [43], and found that miR-29a knockin did not increase the expression of alkaline phosphatase (ALP) in MSC (Figure S2). The present results indicate that the differentiation process is complicated, and mere miR-29a expression cannot completely trigger it.

Previous studies have detected the expression change of miR-29s during the neuronal development [38], [39], whereas, we are the first one to go deeply and discuss the role of miR-29a. This result may be instructive to theoretical research and cellular therapy. MSC is a promising candidate for cell transplantation therapy in neurodegenerative diseases. Genetic manipulation with miR-29a gain-of-function is prospective to promote the production of neurons from MSCs. Previous report proposed that siRNA against REST may be used to produce clinically valid amounts of functional neurons from MSCs [21]. However, it is dangerous in tumor formation, because REST is regarded as a tumor suppressor [44]. The present study showed that miR-29a, also a tumor suppressor [45], may be a safer choice to arrest REST expression and balance the tumor formation in cell therap.

## Conclusions

The present study has demonstrated that the expression of REST decreases upon neuronal differentiation of MSCs, which is partially due to miR-29a upregulation, and miR-29a promotes neuronal differentiation of MSCs through targeting REST, which provides advances in neuronal differentiation research and stem cell therapy for neurodegenerative diseases.

## Supporting Information

Figure S1 **Forced expression of miR-29a in MSCs.** MSCs maintained the normal cell morphology after lentiviral infection of miR-29a precursor. After lentiviral infection of miR-29a precursor or scramble, MSCs expressed EGFP protein. miR-29a knockin did not significantly change cell morphology.
(TIF)

Figure S2 **qRT-PCR results of ALP mRNA expression in MSCs transfected with miR-29a precursor.** The expression of ALP mRNA in MSCs transfected with miR-29a precursor is not different from that in MSCs transfected with scramble. Mean ± SEM of 5 independent MSC cultures are shown.
(TIF)
