# Supplementary figures and images for: miR-29a Modulates Neuronal Differentiation through Targeting REST in Mesenchymal Stem Cells
Source: PLoS One. 2014 May 19;9(5):e97684. doi: 10.1371/journal.pone.0097684 (PMC4026383; doi:10.1371/journal.pone.0097684)

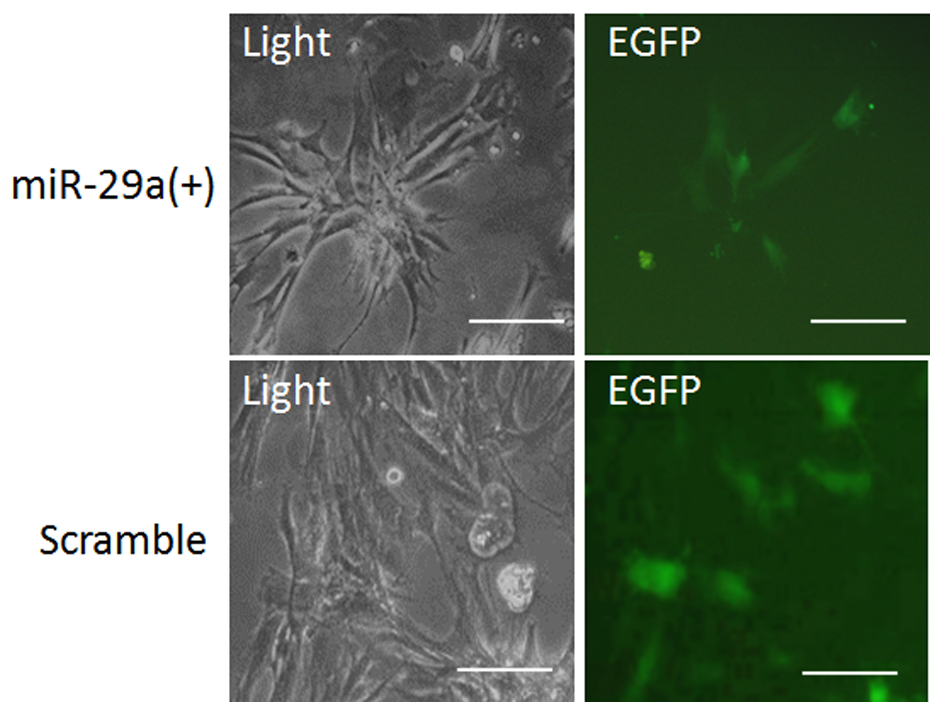

Supplement: Figure S1 — Forced expression of miR-29a in MSCs. MSCs maintained the normal cell morphology after lentiviral infection of miR-29a precursor. After lentiviral infection of miR-29a precursor or scramble, MSCs expressed EGFP protein. miR-29a knockin did not significantly change cell morphology. (TIF) [file pone.0097684.s001.tif]

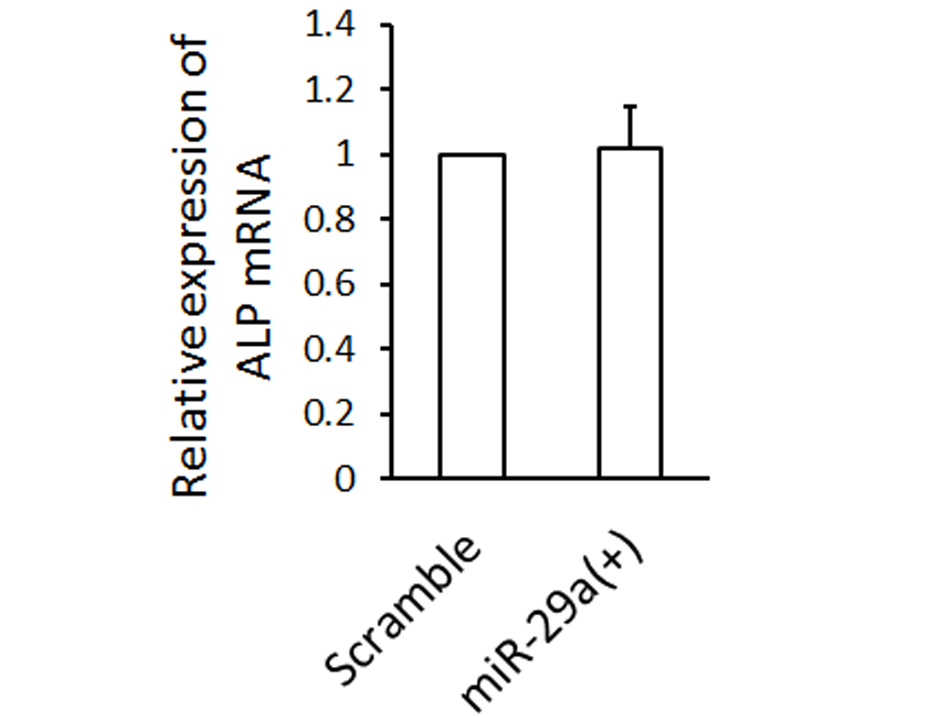

Supplement: Figure S2 — qRT-PCR results of ALP mRNA expression in MSCs transfected with miR-29a precursor. The expression of ALP mRNA in MSCs transfected with miR-29a precursor is not different from that in MSCs transfected with scramble. Mean ± SEM of 5 independent MSC cultures are shown. (TIF) [file pone.0097684.s002.tif]
